# Reliability analysis of radiographic methods for determination of posterolateral lumbossacral fusion

**DOI:** 10.1590/S1679-45082014AO2964

**Published:** 2014

**Authors:** Alberto Ofenhejm Gotfryd, Felipe de Moraes Pomar, Nicola Jorge Carneiro, Fernando José Franzin, Luciano Miller Reis Rodrigues, Patricia Rios Poletto

**Affiliations:** 1 Santa Casa da Misericórdia de Santos, Santos, SP, Brazil.; 2 Faculdade de Medicina do ABC, Santo André, SP, Brazil.; 3 Universidade Federal de São Paulo, Santos, SP, Brazil.

**Keywords:** Spine/radiography, Lumbosacral region/radiography, Arthrodesis/radiography, Bone screws

## Abstract

**Objective:**

To analyze intra and interobserver agreement of two radiographic methods for evaluation of posterolateral lumbar arthrodesis.

**Methods:**

Twenty patients undergoing instrumented posterolateral fusion were evaluated by anteroposterior and dynamic lateral radiographs in maximal flexion and extension. The images were evaluated initially by 6 orthopedic surgeons, and after 8 weeks, reassessed by 4 of them, totaling 400 radiographic measurements. Intra and interobserver reliability were analyzed using the Kappa coefficient and Landis and Koch criteria.

**Results:**

Intra and interobserver agreement regarding anteroposterior radiographs were, respectively, 76 and 63%. On lateral views, these values were 78 and 84%, respectively. However, the Kappa analysis showed poor intra and interobserver agreement in most cases, regardless of the radiographic method used.

**Conclusion:**

There was poor intra and interobserver agreement in the evaluation of lumbosacral fusion by plain film in anteroposterior and dynamic lateral views, with no statistical superiority between the methods.

## RESUMO

**Objetivo:**

Analisar a concordância intra e interobservador de dois métodos radiográficos para avaliação da artrodese lombar posterolateral.

**Métodos:**

Foram submetidos à fusão posterolateral instrumentada 20 pacientes, avaliados por meio de radiografias anteroposteriores e laterais, em flexão e extensão máximas. As imagens foram avaliadas inicialmente por 6 médicos ortopedistas e, após 8 semanas, reavaliadas por 4 deles, totalizando 400 mensurações radiográficas. Foi realizada análise de confiabilidade intra e interobservador por meio do coeficiente Kappa e pelos critérios de Landis e Koch.

**Resultados:**

A porcentagem de concordância intra e interobservadores para radiografias anteroposteriores foi, respectivamente, 76 e 63%. Na incidência radiográfica lateral, esses valores foram de 78 e 84%, respectivamente. Entretanto, a análise pelo método de Kappa mostrou concordância fraca e ruim intra e interobservadores para a maior parte dos casos, independentemente do método radiográfico utilizado.

**Conclusão:**

observou-se fraca concordância intra e interobservadores na avaliação da fusão lombossacra por meio de radiografias simples, nas incidências anteroposterior e laterais dinâmicas, não havendo superioridade estatística entre os métodos estudados.

## INTRODUCTION

Intervertebral fusion is the treatment of choice for symptomatic lumbar instabilities. In most cases, its results are related to the quality of the fusion,^(1,2)^ which makes the imaging method evaluation relevant.

Although surgical exploration is the gold standard for intervertebral fusion determination,^(3)^ the method is no long used routinely, as it is considered too invasive. On the other hand, validity of simple radiography in determining the rate of fusion has been questioned, due to weak interobserver agreement^(4)^ and moderate degree of accuracy (60 and 70%) in determining intervertebral fusion.^(5)^ Even so, radiography is the method most often used for this purpose, due to its availability and low cost.^(6)^

There are different radiographic methods described for the analysis of lumbosacral fusion, such as static anteroposterior, oblique, or dynamic lateral views, in flexion and extension. Each technique has specific characteristics, such as the number of exposures to ionizing radiation, which denotes their safety and costs. Patients submitted to spinal arthrodesis are evaluated by means of radiographs at each clinical visit. For this reason, the number of exposures to ionizing radiation and their diagnostic effectiveness should be optimized.

Computed tomography (CT) is another method described for evaluation of lumbosacral fusion. In the literature, there are few case-control tomographic studies aiming to determine the quality of the intersomatic (anterior) lumbar fusion.^(7–12)^

Currently there is no consensus on the best radiographic method of lumbosacral posterolateral fusion. This observation motivated the present study.

## OBJECTIVE

To analyze the intra and interobserver agreement of two radiographic methods used to evaluate lumbar arthrodesis, by means of static anteroposterior and dynamic lateral radiographies in flexion and extension.

## METHODS

The present study was approved by the Research Ethics Committee of the *Santa Casa da Misericórdia de Santos* (52/10). All the patients accepted to participate and signed the Informed Consent Form (ICF). A cross-sectional study was carried out with 20 patients, 15 of them male. Patients were submitted to instrumented posterolateral lumbar arthrodesis with pedicular screws, operated on between September 2007 and October 2009. Age varied between 43 and 84 years, with a mean of 53.2 years. The minimal postoperative follow-up time was 24 months, with a mean of 32.3 months. The number of anatomical segments operated on varied between 1 and 4, located between L2 and S1.

The inclusion criteria considered patients with degenerative diseases of the spine and mechanical instability; with neural decompression and posterolateral fusion with pedicular screws. Excluded were patients with antecedents of surgery in the lumbar region; metabolic bone disease confirmed by laboratory tests and/or image tests; use of bone substitutes or expanders, and infection at the surgical site with need for surgical cleaning.

In all cases, digital radiographs were obtained of the lumbosacral spine in static anteroposterior views (with 25°caudal inclination of X-ray tube) and in the dynamic lateral views, in maximal flexion and extension. Lateral radiographs were performed with the patient in orthostatic position, with the help of a radiology technician at the time of the test. Radiographic measurements were made by six orthopedic surgeons – three experienced spinal surgeons, two spinal surgery fellows and one orthopedics and traumatology fellow. The cases were initially evaluated by all the examiners. After 8 weeks, radiographic analyses were repeated by 4 examiners, since 2 of the observers were not available for the study. In this way, a total of 400 radiographic measurements were made, and considered for statistical analysis.

**Figure 1:**
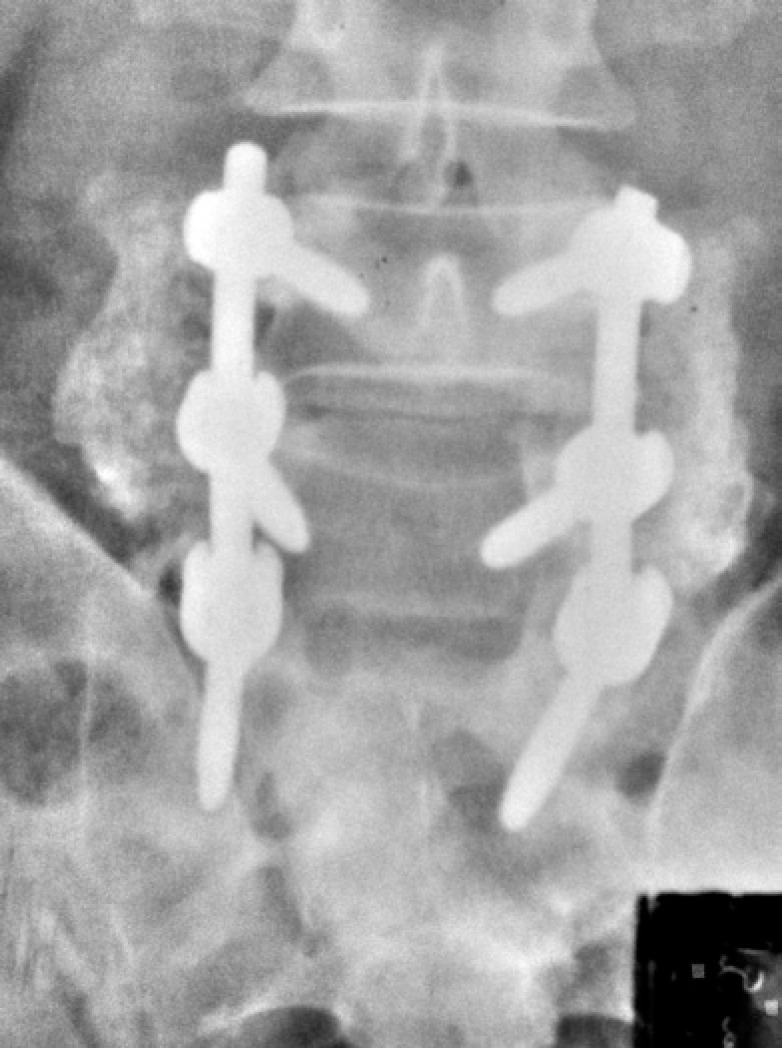
Anteroposterior radiography of the lumbosacral spine showing complete posterolateral fusion

**Figure 2:**
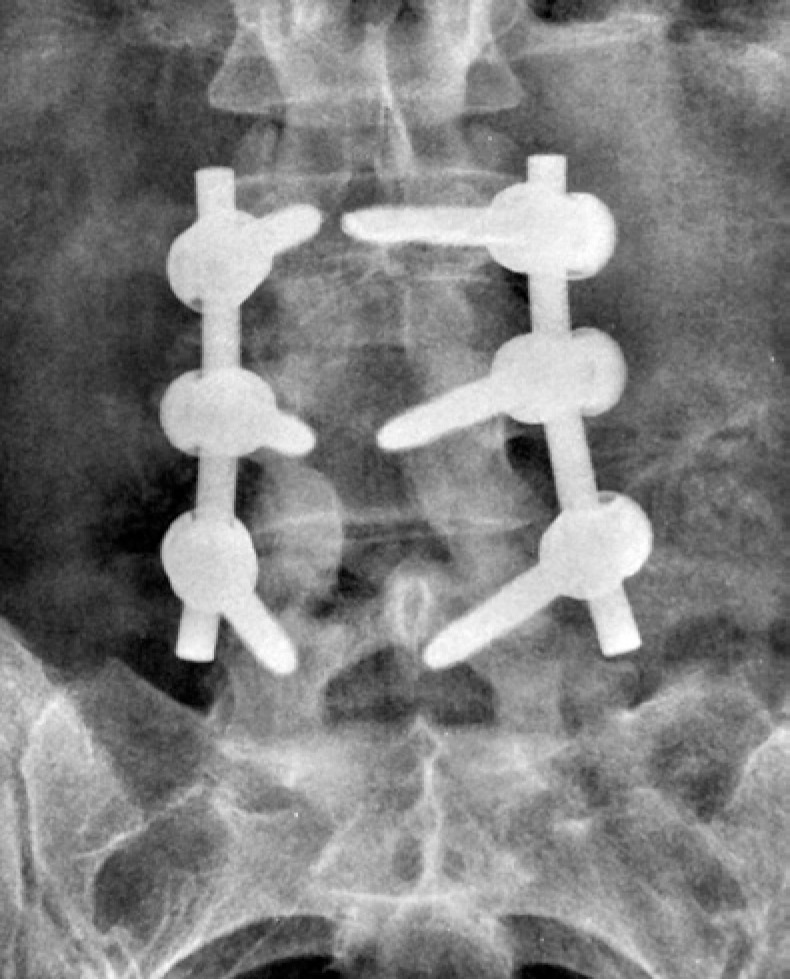
Anteroposterior radiography of the lumbosacral spine displaying doubtful posterolateral fusion, due to the presence of metal stems superimposed on the intertransverse space

**Figure 3:**
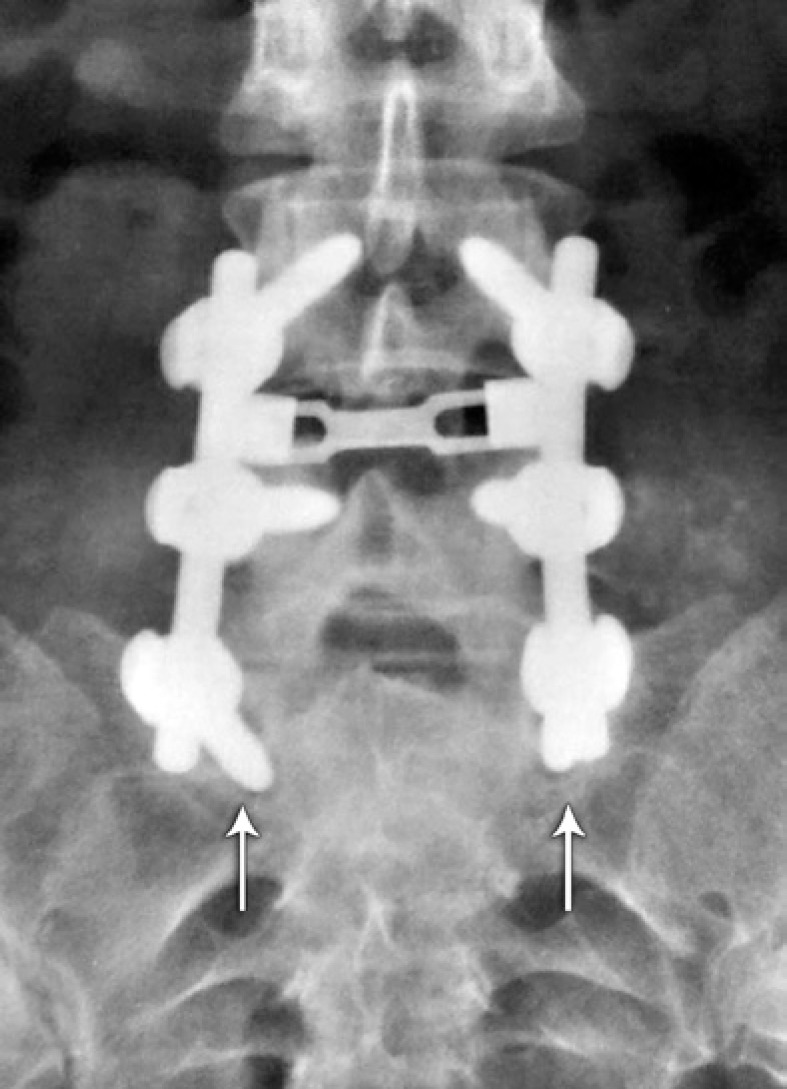
Anteroposterior radiography of the lumbosacral spine, in which pseudoarthrosis is considered due to the presence of osteolysis around screws of S1 (shown by the arrows)

The assessment of fusion from the anteroposterior view followed the criteria proposed by Christensen et al.,^(5)^ that considered fusion positive in the presence of a bone bridge uniting the two adjacent transverse processes, uni- or bilaterally (Figure 1). In cases where overlapping of metal rods precluded an appropriate analysis, the fusion was considered doubtful (Figure 2). Pseudoarthrosis was considered as the absence of a bone bridge between the transverse processes bilaterally (Figure 3).

On the sagittal plane, the criteria of Larsen et al. were used,^(6)^ which consist of the measurement of Cobb's angle between the terminal vertebrae of the fusion. The angles were traced on dynamic radiographies, in maximal flexion and extension of the trunk. Next, the angle difference was calculated between the two measurements. An angle difference ≥5° was considered pseudoarthrosis. The authors also determined lack of fusion the presence of osteolysis greater than 2mm around the pedicular screws, and the breakage or release of the metal implants.

The statistics were performed with the software Statistical Package for the Social Sciences (SPSS, SPSS Inc., Chicago, IL, United States), version 17.0. The descriptive analysis is shown in percentage of agreement among observers and intraobserver. Reliability analysis was carried out using Kappa coefficient, with values of −1 to 1, where those near “one” are considered in greater agreement. The result analysis, after the Kappa coefficient calculation, was interpreted according to criteria proposed by Landis and Koch.^(13)^

## RESULTS

### Anteroposterior radiograph

The interobserver reproducibility (Table 1) was evaluated comparing the first reading of each observer, paired two by two, in order to cover all the possible combinations analyzed by the Kappa coefficient. In this analysis, the mean percentage of agreement was 76% (standard deviation of 7.8).

**Table 1:** Results of interobserver reliability for anteroposterior radiographs

Observers	Agreement (%)	Kappa	Reliability level
Observer 1×2	82	0.32	Reasonable
Observer 1×3	77	0.24	Reasonable
Observer 1×4	86	0.32	Reasonable
Observer 1×5	86	0.50	Moderate
Observer 1×6	68	0.12	Poor
Observer 2×3	77	0.29	Reasonable
Observer 2×4	78	0.33	Reasonable
Observer 2×5	77	0.36	Reasonable
Observer 2×6	84	0.36	Reasonable
Observer 3×4	64	0.10	Poor
Observer 3×5	74	0.21	Reasonable
Observer 3×6	72	0.18	Poor
Observer 4×5	64	0.07	Poor
Observer 4×6	86	0.34	Reasonable
Observer 5×6	64	0.07	Poor

Mean percentage of agreement: 76% (standard deviation 7.8).

The intraobserver reproducibility (Table 2) study using the Kappa coefficient showed a mean percentage of agreement of 63% (standard deviation of 10).

**Table 2:** Intraobserver reliability analysis results for anteroposterior radiographies

Observers	Agreement (%)	Kappa	Reliability level
Observer 3	55	0.06	Poor
Observer 4	70	0.26	Reasonable
Observer 5	73	0.19	Poor
Observer 6	53	0.09	Poor

Mean percentage of agreement: 63% (standard deviation of 10).

### Evaluation of dynamic lateral radiographies

The study of interobserver reproducibility (Table 3) was conducted by comparing the first reading of each observer, paired two by two, in order to cover all the combinations possible analyzed by Kappa's coefficient. The mean percentage of agreement was 78% (standard deviation of 9.1).

**Table 3:** Interobserver reliability analysis results for dynamic lateral incidences

Observers	Agreement (%)	Kappa	Reliability level
Observer 1×2	66	0.01	Poor
Observer 1×3	84	-0.08	Poor
Observer 1×4	86	0.18	Poor
Observer 1×5	89	0.39	Reasonable
Observer 1×6	82	0.26	Reasonable
Observer 2×4	84	0.27	Reasonable
Observer 2×5	77	0.35	Reasonable
Observer 2×6	70	0.18	Poor
Observer 3×2	64	0.01	Poor
Observer 3×4	61	0.02	Poor
Observer 3×5	82	0.10	Poor
Observer 3×6	89	0.38	Reasonable
Observer 4×5	86	0.50	Moderate
Observer 4×6	80	0.21	Reasonable
Observer 5×6	75	0.12	Poor

Mean percentage of agreement: 78% (standard deviation of 9.1).

The interobserver reliability study (Table 4), performed with four observers (Kappa coefficient) showed a mean percentage of agreement of 84% (standard deviation of 10).

**Table 4:** Intraobserver reliability analysis results for dynamic lateral views

Observers	Agreement (%)	Kappa	Reliability level
Observer 3	95	0.73	Substantial
Observer 4	82	0.41	Moderate
Observer 5	82	0.23	Reasonable
Observer 6	75	0.20	Reasonable

Mean percentage of agreement: 84% (standard deviation of 10).

## DISCUSSION

Intervertebral fusion is a common procedure to treat mechanical instabilities of the spine. There are several operative techniques for that purpose and for all, a solid fusion among the adjacent vertebrae represents a primary outcome, so that improves clinical results.^(14,15)^ Posterolateral arthrodesis is a relatively low-cost method (when compared to other instrumented techniques) and simple for spinal surgeons to perform. The technique is popular among orthopedic surgeons and neurosurgeons, and for these reasons, was chosen for analysis in the present study.

There is no consensus on the best radiographic way to evaluate the quality of lumbar vertebral fusion. Imaging methods include radiography and tomography, but the final diagnosis is confirmed by surgical inspection.^(3,6,14)^ Blumenthal et al.^(16)^ assessed 49 patients with lumbar fusion and compared the radiographic results with those obtained surgically. The authors observed a clinical-radiographic correlation in merely 59% of cases. However, for obvious reasons, operative exploration is reserved for those patients with unsatisfactory clinical results and high radiographic suspicion of fusion failure. For the rest, the need for adequate evaluation by imaging studies is imperative. Currently, there is no scientific evidence of a lumbar fusion diagnostic method superior to the others.

In this study, for the analysis of lumbar fusion two radiographic methods were compared. The first, proposed by Christensen et al.,^(5)^ is based on a “quadrant” classification system, in which each intertransverse space is subdivided into units to be applied separately. Fusion is considered as the presence of a bone bridge uniting the transverse processes bilaterally or unilaterally at each anatomic level. In the original article, the authors describe inter- and intraobserver agreement of 86 and 93%, respectively. The present study revealed values of 76 and 63%, respectively, and inter- and intraobserver agreement was considered weak for most of the cases studied. One possible explanation for this may consist in the fact of no bowel preparation having been made before the radiographic study, a fact that may have contributed towards worsening of image quality, leading to interpretation error. For outpatient reevaluation of lumbar fusions, however, the use of simple radiographs with no bowel preparation is a common practice among spinal surgeons.

A study by means of dynamic lateral radiographs, in maximal flexion and extension, was also performed. It is believed that there should be minimal movement between the two adequately fused vertebrae.^(17–20)^ Larsen et al. demonstrated the existence of residual mobility after lumbar fusion – evaluated by dynamic radiographs –and that these could vary according to the type of fusion performed (posterolateral, intersomatic, or anterior).^(6)^

In this study, we used the criteria proposed by the referred authors who proposed pseudoarthrosis as the presence of an angle difference ≥5° among the instrumented vertebrae views on the dynamic lateral radiographs. With this method, as an example of what was cited above, poor or weak intra- and interobserver agreement was observed for most of the cases studied. In the results found, no influence was noted of time of professional experience of the observing doctors.

Rodrigues et al.^(21)^ demonstrated that the presence of lumbar pseudoarthrosis did not promote worse clinical results than those observed in patients with complete fusion. These findings are similar to those described by Kant et al.,^(14)^ that suggested that the concern with the quality of fusion is more relevant only in those patients with an unfavorable clinical result and chronic lumbar pain.

The minimal radiographic study of the spine should include two orthogonal views.^(22)^ Besides these, additional views, oblique^(23)^ or dynamic, may offer more information as to presence of fusion mass. This, however, increases exposure to ionizing radiation as well as financial costs. It is estimated that each simple X-ray of the lumbar spine furnished approximately 1.5mSv of radioactive load to the patient.^(24)^ This quantity is equivalent to about 75 simple chest X-rays, which would have an approximate radiation load of about 0.02mSv. In this way, patients evaluated by simple X-rays by Christensen's method received about 1.5mSv per test, while dynamic radiography (two radiographic views) offered an exposure of 3mSv (equivalent to 150 simple chest X-rays). As to dynamic lateral radiographies in flexion and extension, the rationale of the method is questioned. In the present study, no additional information was observed as to intervertebral fusion when compared to the anteroposterior view (a single radiographic exposure).

The use of CT in the evaluation of lumbosacral fusion is described in the literature. The method is frequently used to verify intersomatic (interbody) fusion. In the present study, patients were submitted exclusively to posterolateral fusion, with no addition of intersomatic grafts.

Currently, the tomographic studies available are of the case-control or case series types. Rothman et al. recommended the use of CT to evaluate anterior fusion of the spine.^(7)^ Other authors also demonstrated superiority of CT relative to dynamic radiographs for the determination of intersomatic lumbosacral fusion. ^(8,10,11)^ However, these findings diverge from the results by Fogel et al., who demonstrated the tomographic study is unnecessary in cases with signs of pseudoarthrosis on plain X-ray films.^(9)^ Although not the objective of this study, it is believed that CT might be considered an additional method for determining the existence of lumbosacral fusion mass, especially in patients that present with unsatisfactory surgical results and with a suspicion of consolidation failure.

## CONCLUSION

A weak intra-and interobserver agreement in lumbosacral fusion by means of plain-film radiography was noted, in anteroposterior and dynamic lateral views, with no statistical superiority between the studies.

## References

[1] Kirkpatrick JS, Scholl BM, Frymoyer JW, Wiesel SW (2004). Posterolateral lumbar fusion. The adult and pediatric spine.

[2] Heggeness MH, Esses SI (1991). Classification of pseudarthroses of the lumbar spine. Spine (Phila Pa 1976).

[3] Brodsky A, Kovalsky E, Khalil M (1991). Correlation of radiologic assessment of lumbar spine fusions with surgical exploration. Spine (Phila Pa 1976).

[4] Laasonen EM, Soini J (1989). Low-back pain after lumbar fusion. Surgical and computed tomographic analysis. Spine (Phila Pa 1976).

[5] Christensen FB, Laursen M, Gelineck J, Eiskjaer SP, Thomsen K, Bünger CE (2001). Interobserver and intraobserver agreement of radiograph interpretation with and without pedicle screw implants: the need for a detailed classification system in posterolateral spinal fusion. Spine (Phila Pa 1976).

[6] Larsen JM, Rimoldi RL, Capen DA, Nelson RW, Nagelberg S, Thomas JC (1996). Assessment of pseudoarthrosis in pedicle screw fusion: a prospective study comparing plain radiographs, flexion/extension radiographs, CT scanning and bone scintigraphy with operative findings. J Spinal Disord.

[7] Rothman SL, Glenn WV (1985). CT evaluation of interbody fusion. Clin Orthop Relat Res.

[8] Shah RR, Mohammed S, Saifuddin A, Taylor BA (2003). Comparison of plain radiographs with CT scan to evaluate interbody fusion following the use of titanium interbody cages and transpedicular instrumentation. Eur Spine J.

[9] Fogel GR, Toohey JS, Neidre A, Brantigan JW (2008). Fusion assessment of posterior lumbar interbody fusion using radiolucent cages: X-ray films and helical computed tomography scans compared with surgical exploration of fusion. Spine J.

[10] Lee HS, Lee JH, Lee JH (2013). A comparison of dynamic views using plain radiographs and thin-section three-dimensional computed tomography in the evaluation of fusion after posterior lumbar interbody fusion surgery. Spine J.

[11] Santos ER, Goss DG, Morcom RK, Fraser RD (2003). Radiologic assessment of interbody fusion using carbon fiber cages. Spine (Phila Pa 1976).

[12] Nakashima H, Yukawa Y, Ito K, Horie Y, Machino M, Kanbara S (2011). Extension CT scan: its suitability for assessing fusion after posterior lumbar interbody fusion. Eur Spine J.

[13] Landis JR, Koch GG (1977). The measurement of observer agreement for categorical data. Biometrics.

[14] Kant AP, Daum WJ, Dean SM, Uchida T (1995). Evaluation of lumbar spine fusion. Plain radiographs versus direct surgical exploration and observation. Spine (Phila Pa 1976).

[15] Watkins MB (1953). Posterolateral fusion of the lumbar and lumbosacral spine. J Bone Joint Surg Am.

[16] Blumenthal SL, Gill K (1993). Can lumbar spine radiographs accurately determine fusion in postoperative patients? Correlation of routine radiographs with a second surgical look at lumbar fusions. Spine (Phila Pa 1976).

[17] Carreon LY, Djurasovic M, Glassman SD, Sailer P (2007). Diagnostic accuracy and reliability of fine-cut CT scans with reconstructions to determine the status of an instrumented posterolateral fusion with surgical exploration as reference standard. Spine (Phila Pa 1976).

[18] Resnick DK, Choudhri TF, Dailey AT, Groff MW, Khoo L, Matz PG, Mummaneni P, Watters WC, Wang J, Walters BC, Hadley MN, American Association of Neurological Surgeons/Congress of Neurological Surgeons (2005). Guidelines for the performance of fusion procedures for degenerative disease of the lumbar spine. Part 4: radiographic assessment of fusion. J Neurosurg Spine.

[19] Stauffer RN, Coventry MB (1972). Posterolateral lumbar-spine fusion. Analysis of the Mayo Clinic Series. J Bone Joint Surg.

[20] Bjarke Christensen F, Stender Hansen E, Laursen M, Thomsen K, Bünger CE (2002). Long-term functional outcome of pedicle screw instrumentation as a support for posterolateral spinal fusion: randomized clinical study with a 5-year follow up. Spine.

[21] Rodrigues LM, Ueno FH, Fujiki EN, Milani C (2011). Estudo prospectivo comparativo entre pseudartrose e fusão óssea na estenose de canal lombar. Acta Ortop Bras.

[22] Turner JA, Herron L, Dayo RA (1993). Meta-analysis of the results of lumbar spine fusion. Acta Orthop Scand Suppl.

[23] Ebraheim NA, Xu R (1998). Assessment of lumbosacral fusion mass by angled radiography. Technical notes. Spine (Phila Pa 1976).

[24] Mettler FA, Huda W, Yoshizumi TT, Mahesh M (2008). Effective doses in radiology and diagnostic nuclear medicine: a catalog. Radiology.

